# Biomarker identification in serum samples from patients with recurrent cervical cancer treated with ADXS11-001 immunotherapy

**DOI:** 10.1186/2051-1426-1-S1-P61

**Published:** 2013-11-07

**Authors:** Poonam Molli, Inga Malinina, Anu Wallecha

**Affiliations:** 1 Research and Development, Advaxis, Inc., Princeton, NJ, USA

## 

Cervical Cancer is the third most common cancer worldwide and is almost always caused by human papilloma virus (HPV) infection. The HPV genes E6 and E7 are known to be oncogenes that promote cellular changes in infected cells leading to malignant transformation. We have previously reported that ADXS11-001 immunotherapy, a live attenuated Listeria monocytogenes (Lm), bioengineered to secrete a LLO-E7 fusion protein, can be safely administered to patients with late stage cervical cancer. In a Phase 2 study being conducted in India, 110 patients with recurrent cervical cancer received 264 doses of ADXS11-001. In this study, serum samples were collected from each patient at three different time points: pre-dose; 2h and 4h post-dose of ADXS11-001 immunotherapy. The initial analysis of 18 serum samples using 45-biomarker multi-analyte human inflammation MAPv1.0 (Myriad RBM) showed differential increases in cytokine and chemokine levels in each sample at 2 and 4 hours post-dosing with ADXS11-001. More than a 15-fold increase was observed in the level of cytokines (IL-6, IL-8, IL-10 and TNF-α) and chemokines (MIP-1α, MIP-1β and MCP-1), indicating strong stimulation of innate immunity. We will report our evaluation of RNA from serum samples collected from patients at pre- and post-dosing of ADXS11-001 using a genomics-based approach and evaluate correlation with changes in cytokine and chemokine levels. We will further report the association of changes in the expression of cytokines or other serum factors with the severity of adverse events as well as clinical responses. In conclusion, RNA or protein expression profiles from patient serum may help in identifying biomarkers which may be employed as a screening tool in future studies to predict efficacy, monitor side effects, and suggest more precise toxicity management for patients treated with of ADXS11-001 immunotherapy.

